# Muscle Dysfunction and Bone Loss in a Woman With Cystic Fibrosis and Obesity Treated With Glucagon‐Like Peptide 1 Agonist: A Case Report

**DOI:** 10.1002/rcr2.70541

**Published:** 2026-03-10

**Authors:** Shanal Kumar, Robyn Cobb, Angela Matson, Joseph Lee, Daniel Henderson

**Affiliations:** ^1^ The Adult Cystic Fibrosis Centre (ACFC), the Prince Charles Hospital, Chermside Queensland Chermside Australia; ^2^ The University of Queensland St Lucia Queensland Australia; ^3^ Dept Physiotherapy Allied Health the Prince Charles Hospital, Chermside Queensland Chermside Australia; ^4^ Dept Nutrition & Dietetics Allied Health the Prince Charles Hospital, Chermside Queensland Chermside Australia; ^5^ Dept Radiology, the Prince Charles Hospital, Chermside Queensland Chermside Australia

**Keywords:** bone, cystic fibrosis, diabetes, glucagon like peptide 1 agonist, muscle, obesity

## Abstract

Cystic fibrosis (CF) modulator therapies can lead to rapid and excessive weight gain. Obesity in CF can lead to undesirable metabolic complications including type 2 diabetes. Therefore, glucagon‐like peptide 1 (GLP‐1) agonists which can facilitate weight loss and improve metabolic profiles are increasingly prescribed to people with CF. We present the first case report documenting serial changes in body composition and bone mineral density over a 3‐year period in an adult with CF, obesity and diabetes treated with Semaglutide and Elexacaftor/Tezacaftor/Ivacaftor (ETI). Our case experienced substantial weight gain following ETI initiation which exacerbated hyperglycaemia. This prompted initiation of Semaglutide, which over 12 months led to 14.8% total body weight loss and significant glycaemic improvement. However, serial assessments revealed declines in absolute muscle mass, muscle strength and bone mineral density despite prescription of tailored exercise and nutritional interventions. Our findings highlight the need for cautious use of GLP‐1 in people with CF.

## Graphical abstract

Cystic fibrosis (CF) modulator therapies can lead to rapid and excessive weight gain. Obesity in CF can lead to undesirable metabolic complications including type 2 diabetes. Therefore, glucagon‐like peptide 1 (GLP‐1) agonists which can facilitate weight loss and improve metabolic profiles are increasingly prescribed to people with CF. We present the first case report documenting serial body composition and BMD changes in an adult with CF, obesity and diabetes receiving Semaglutide and highlight concerning losses in bone mass and muscle function despite metabolic benefits.

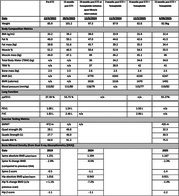

## 1 Introduction

Weight gain and metabolic complications are emerging concerns in adults with cystic fibrosis (CF) following introduction of Elexacaftor/Tezacaftor/Ivacaftor (ETI). Glucagon‐like peptide 1 agonists (GLP‐1) are gaining interest as treatment for obesity and diabetes [1] with potential lung benefits [2]. Prevalence of GLP‐1 use may be as high as 6.8% of the CF population [3, 4]. However, their impact on body composition, specifically bone mineral density (BMD) and muscle mass/function, which are intricately coupled [5] remains poorly described in the literature. We present the first case report documenting serial body composition and BMD changes in an adult with CF, obesity, and diabetes receiving Semaglutide and highlight concerning losses in bone mass and muscle function despite metabolic benefits.

## 2 Case Report

A 42‐year‐old pre‐menopausal woman, heterozygous for F508del with mild lung disease and pancreatic insufficiency with stable nutritional status was referred to our CF Diabetes Support service. Her history included depression, gestational diabetes in 2010 (requiring insulin) and a strong paternal family history of type 2 diabetes.

In 2018, a screening oral glucose tolerance test (5.9 | 10.9 | 12.7 mmol/L) confirmed diabetes, with a contemporaneous Haemoglobin A1c (HbA1c) of 6.5% (48 mmol/mol). She experienced significant needle phobia and initially engaged with a regional non‐CF provider, commencing Metformin 1 g/Sitagliptin 50 mg daily for her diabetes management.

At ETI initiation in December 2022, her HbA1c was 7.3% (56 mmol/mol) on triple combination oral therapy (Metformin 1000 mg BD, Gliclazide 30 mg daily and Sitagliptin 50 mg daily). She weighed 86.3 kg (BMI 33.1 kg/m^2^) with 46% body fat and 50.1% muscle mass—See Table 1. She reported increased energy levels and engagement in incidental physical activity after starting ETI.

**TABLE 1:** Changes in weight, body composition, lung function, exercise testing metrics and bone mineral density over 3 years following sequential initiation of ETI and GLP‐1 agonist.

	Pre‐ETI	12‐months post ETI	18‐months post ETI at Semaglutide initiation (post lifestyle intervention)	3‐months post ETI + Semaglutide	8‐months post ETI + Semaglutide	12‐months post ETI + Semaglutide
Date	12/4/2022	28/6/2023	12/6/2024	11/9/2024	12/2/2025	6/08/2025
Weight	85.9	101.2	97.3	87.9	82.8	82.9 kg
Body Composition Metrics						
BMI kg/m2	33.2	39.2	38.0	33.9	31.9	32.4
Fat %	46.0	50.1	47.0	44.6	42.6	41.5
Fat mass (kg)	39.8	51.6	45.7	39.2	35.3	34.4
Muscle %	51.2	46.5	50.4	52.6	54.3	55.5
Muscle mass (kg)	44.0	47.1	49	46.2	45.1	46
Total Body Water (TBW) (kg)	n/a	n/a	36	34.2	34.8	34.0
TBW %	n/a	n/a	37	38.9	42	41
Bone mass (kg)	2.5	2.5	2.6	2.5	2.4	2.5
BMR (kJ)	n/a	n/a	6770	6343	6150	6247
BMR (calories)	n/a	n/a	1618	1516	1470	1492
Blood pressure (mmHg)	115/82	111/80	118/79	n/a	115/77	119/82
Lung Function						
ppFEV1	37.30%	53.73%	n/a		n/a	55.27%
FEV1	1.08 L	1.54 L			1.56 L	n/a
FVC	1.83 L	2.46 L	n/a		2.96 L	n/a
Exercise Testing Metrics						
6MWT	472 m	n/a	n/a			435 m
Quad strength (R)	28.1	46.8				32.3
Quads Strength (L)	27.7	46.0				30.3
Quads BW %	64.9	91.3				75.5
Bone Mineral Density (from Dual X‐ray Absorptiometry [DXA])						
Date	2019		2024			2025
Spine absolute BMD g/per/sqcm	1.231		1.194			1.167
Spine % change BMD (compared to previous DXA)	−2.9%		−0.5%			−2.3%
Spine Z‐score	−0.5		−1.1			−1.4
Hip absolute BMD g/per/sqcm	1.016		0.943			0.921
Hip % change BMD (c/w previous scan)	+1.1%		−7.2%			−2.3%
Hip Z‐score	−1.1		‐ 2.1			−2.2

One‐year post‐ETI, her weight peaked at 101.2 kg (Δ +15.0 kg, BMI 39.2 kg/m^2^) coinciding with a HbA1c of 10.1% (87 mmol/mol). She presented with a skin abscess requiring surgical drainage prompting referral back to our CF Diabetes support services. She described symptoms of hyperglycaemia, recurrent episodes of vulvovaginal candidiasis, intense sugar cravings and significant distress over her rapid weight gain. Her sodium glucose co‐transporter 2 inhibitor commenced by her regional non‐CF providers was ceased with immediate resolution of vulvovaginal candidiasis.

With guided lifestyle interventions mostly focused on reducing intake of ultra‐processed foods, she lost 3.8 kg over three months. Following this, comprehensive consultations were conducted incorporating nutritional advice to reduce excess energy from discretionary foods and include a balanced dietary intake to avoid malnutrition, plus engagement in strength training to mitigate muscle loss. This included a personalised exercise plan. Following this multidisciplinary consultation, Sitagliptin was ceased, and weekly 0.25 mg Semaglutide, a GLP‐1 agonist, commenced in June 2024.

She experienced moderate gastrointestinal side effects, specifically dyspepsia, nausea, and vomiting during the initiation phase for up to six weeks. This was managed with dietary adjustments, hydration, aperients, and proton pump inhibitor use. On Semaglutide 0.5 mg weekly, mild side effects persisted.

After three months, she had lost 9.4 kg (approximately 0.8 kg per week) with reductions in absolute fat, muscle and bone mass (See Table 1). Continuous glucose monitoring showed 88% time spent in range (3.9–10 mmol/L, 70–180 mg/dL), a mean glucose of 8.2 mmol/L (148 mg/dL) and HbA1c at target. Gliclazide was ceased and she was continued on Metformin 1 g twice daily and Semaglutide 0.5 mg weekly.

By six months, her weight loss had plateaued, sugar cravings returned, and strength training adherence waned, compounded by psychosocial stressors and a predominantly sedentary lifestyle. Semaglutide was increased to 1 mg weekly, and she was commenced on a multivitamin and calcium supplementation. Within two weeks of the higher dose, she developed moderate gastrointestinal side effects and mild dehydration, but investigations were unremarkable and treatment was continued.

At nine months, she had lost an additional 5.1 kg in weight. She described ongoing issues with sugar cravings, for which she was provided psychological and dietetic support. Her HbA1c remained at target; however, serial body composition revealed declining muscle mass and BMD, despite stable percentage muscle mass. Recommendations to maintain strength training, multivitamin, calcium, and vitamin D supplementation were reiterated.

At 12‐months post initiation of Semaglutide, total weight loss was 14.4 kg (Δ −14.8% total body weight). Serial dual‐energy X‐ray Absorptiometry (DXA) demonstrated declines in BMD at the spine and the hip with Z‐scores falling into the osteopenic (−1.4) and osteoporotic (−2.2) range, respectively. Prior to ETI initiation, her Z‐scores were stable in the normal range at −0.5 and −1.1 at the spine and hip, respectively.

She had regular menses, replete vitamin D and an unremarkable secondary osteoporosis screen which comprised evaluation for thyroid and parathyroid dysfunction, coeliac disease, multiple myeloma, calcium and phosphate disorders. Functional testing including quadriceps strength testing with the use of a dynamometer confirmed loss of muscle strength. This was also reflected in decreased aerobic exercise capacity distance with the six‐minute walk test. (See Table 1). Serial basal metabolic rates declined consistent with counter‐regulatory adaptations. She was counselled on strength training, fracture prevention and continued calcium/vitamin D supplementation.

The patient reported an overall positive experience with Semaglutide therapy, associated metabolic benefits and the multidisciplinary clinical support that she received. However, she also acknowledged our concerns regarding muscle dysfunction and bone loss, particularly given her age and impending peri‐menopause. A shared decision to continue Semaglutide 1 mg weekly was made, with psychology support to address her sugar cravings and barriers to lifestyle optimisations, particularly exercise engagement. She was also receptive to information on pharmacotherapy, which may be required to support her bone health in the near future.

## 3 Discussion

To the best of our knowledge, this is the first case report tracking bone and muscle changes in an adult with CF, obesity and diabetes during GLP‐1 agonist therapy. Over 12 months, Semaglutide produced substantial weight loss and glycaemic improvement but was accompanied by measurable declines in absolute muscle mass, muscle strength and BMD.

Weight gain following ETI initiation has been documented, with gains in visceral adiposity driving insulin resistance and metabolic syndrome in CF an emerging concern [6]. In our case, rapid post‐ETI weight gain coincided with deteriorating glycaemia. Semaglutide optimised glycaemia likely through augmented glucose‐dependent endogenous insulin secretion [7], however, declines in muscle function and BMD were concerning additional findings.

In the general population with normal ageing, approximately 1% of skeletal muscle atrophies annually from middle age [8]. Muscle loss can lead to declining physical function and conditioning leading to frailty, increased risk of falls and fracture [9]. Sarcopenia, defined as muscle deficiency, has also been linked to higher all‐cause mortality [9]. During weight loss, resistance training has been shown to preserve muscle mass [10]. There is also emerging evidence of peripheral muscle dysfunction in people with CF, which is likely multi‐factorial and related to physical inactivity, inflammation, metabolic abnormalities and malnutrition [11]. Additionally, emerging evidence suggests ETI‐related muscle gains may be limited [12]. Our case experienced a decline in muscle function despite ETI adherence and targeted support in the form of telehealth engagement, home exercise prescriptions, referrals to local exercise physiologist and continued MDT education. We postulate her sedentary behaviour due to underlying CF was further exacerbated by GLP‐1 therapy, which perhaps limited her capacity to engage in prescribed exercise interventions. Our case highlights the complexities of attempting to preserve and/or maximise muscle health and function in people with CF, especially during a weight loss phase and with ageing.

Bone loss is another concern in CF, where low BMD is prevalent. In our case, BMD declined from near‐normal pre‐ETI to the osteoporotic range at the hip over 2.5 years in the setting of weight loss. This is likely to have been compounded by a sedentary lifestyle, adherence issues to prescribed nutritional and exercise interventions, and rapid weight loss. Our findings underscore the potential vulnerability of bone health in the CF population with weight loss. This might be particularly relevant for women living with CF, who may face accelerated bone loss during pregnancy, peri‐menopause, and post‐menopause. Evidence indicates exercise can mitigate some of these negative effects and promote long‐term weight management after weight loss [13], however, limitations imposed by CF‐related muscle dysfunction may need to be considered.

In our case, despite delivery of personalised dietetic, exercise and diabetes supports through a co‐designed multi‐disciplinary model‐of‐care [14], we were unable to completely mitigate adverse musculoskeletal treatment‐related outcomes during GLP‐1 therapy and associated weight loss. While use of GLP‐1 therapies can be useful for the management of diabetes and obesity in CF, our findings suggest a need for baseline and serial monitoring of body composition. Recently published guidance on exercise testing will also assist standardised musculoskeletal assessments [15]. Our case report also highlights the need for more research into musculo‐skeletal complications associated with weight loss in adults with CF and obesity.

## Author Contributions

**Shanal Kumar:** conceptualized this manuscript, managed project administration including data collection, and wrote the original draft. **Angela Matson:** data collection, provided critical reviewing and editing. **Robyn Cobb:** data collection, providing critical reviewing and editing. **Joseph Lee:** data collection, provided critical reviewing and editing, supervision. **Daniel Henderson:** data collection, provided critical reviewing and editing, supervision.

## Funding

S.K. is supported by Queensland Health Clinical Research Fellowship.

## Consent

The authors declare that written informed consent was obtained for the publication of this manuscript and accompanying images and attest that the form used to obtain consent from the patient complies with the Journal requirements as outlined in the author guidelines.

## Conflicts of Interest

The authors declare no conflicts of interest.

## Data Availability

The data that support the findings of this study are available from the corresponding author upon reasonable request.
